# State-level clustering in PrEP implementation factors among family planning clinics in the Southern United States

**DOI:** 10.3389/fpubh.2023.1214411

**Published:** 2023-07-25

**Authors:** Anandi N. Sheth, Kimberly P. Enders, Micah McCumber, Matthew A. Psioda, Aditi Ramakrishnan, Jessica M. Sales

**Affiliations:** ^1^Department of Medicine, Division of Infectious Diseases, Emory University School of Medicine, Atlanta, GA, United States; ^2^Department of Biostatistics, University of North Carolina at Chapel Hill, Chapel Hill, NC, United States; ^3^Department of Behavioral, Social, and Health Education Sciences, Rollins School of Public Health, Emory University, Atlanta, GA, United States

**Keywords:** Southern U.S., women, PrEP, family planning, implementation

## Abstract

**Background:**

Availability of PrEP-providing clinics is low in the Southern U.S., a region at the center of the U.S. HIV epidemic with significant HIV disparities among minoritized populations, but little is known about state-level differences in PrEP implementation in the region. We explored state-level clustering of organizational constructs relevant to PrEP implementation in family planning (FP) clinics in the Southern U.S.

**Methods:**

We surveyed providers and administrators of FP clinics not providing PrEP in 18 Southern states (Feb-Jun 2018, *N* = 414 respondents from 224 clinics) on these constructs: readiness to implement PrEP, PrEP knowledge/attitudes, implementation climate, leadership engagement, and available resources. We analyzed each construct using linear mixed models. A principal component analysis identified six principal components, which were inputted into a K-means clustering analysis to examine state-level clustering.

**Results:**

Three clusters (C1–3) were identified with five, three, and four states, respectively. Canonical variable 1 separated C1 and C2 from C3 and was primarily driven by PrEP readiness, HIV-specific implementation climate, PrEP-specific leadership engagement, PrEP attitudes, PrEP knowledge, and general resource availability. Canonical variable 2 distinguished C2 from C1 and was primarily driven by PrEP-specific resource availability, PrEP attitudes, and general implementation climate. All C3 states had expanded Medicaid, compared to 1 C1 state (none in C2).

**Conclusion:**

Constructs relevant for PrEP implementation exhibited state-level clustering, suggesting that tailored strategies could be used by clustered states to improve PrEP provision in FP clinics. Medicaid expansion was a common feature of states within C3, which could explain the similarity of their implementation constructs. The role of Medicaid expansion and state-level policies on PrEP implementation warrants further exploration.

## Introduction

Despite advances in HIV treatment and prevention technologies, HIV continues to be a persistent public health issue in the United States (U.S.) that particularly affects minoritized and marginalized communities resulting in significant HIV health disparities. Black Americans comprise only 13% of the U.S. population, yet account for roughly 40% of new HIV diagnoses (1). In 2020, the ten states with the largest non-Hispanic black population (Alabama, Arkansas, Delaware, Georgia, Louisiana, Maryland, Mississippi, North Carolina, South Carolina, and Virginia) were all located in the U.S. Department of Health and Human Services (DHHS) Regions III, IV and VI, which collectively encompass the U.S. (2) South. Racialized HIV disparities are pronounced in Southern states where the current HIV epidemic is highly concentrated, with nearly 60% of new HIV diagnoses occurring among Black individuals. Though the highest HIV infection rates in the U.S. continue to occur in Black men who have sex with men, in 2021, cis-gender women accounted for 18% of all new HIV infections nationally, among which 54% are Black cis-gender women (3).

In 2019, the U.S. Department of Health and Human Services’, *Ending the HIV Epidemic (EHE): A Plan for America*, identified 4 key pillars (Diagnosis, Prevent, Treat, and Respond) to achieve an end to the HIV epidemic in the U.S. by 2030 (4). Since then, U.S. federal agencies have been working in a coordinated manner, with their initial focus on vulnerable populations (e.g., Black women) and geographic hotspots (e.g., Southern states/jurisdictions). The primary emphasis of the Prevent pillar includes prioritization of biomedical prevention tools like HIV pre-exposure prophylaxis (PrEP) that reduce HIV transmission up to 99% with consistent use (5). However, in 2021, cis-gender women represented only 8% of PrEP users in the U.S. despite comprising 18% of new HIV diagnoses (6). PrEP use has increased among men, but remains flat in women (7), with disproportionately low use among women in the Southern U.S. and Black and Hispanic women specifically (8). Two prominently reported reasons for low PrEP uptake among women in the US, and particularly in the Southern U.S., have been women’s lack of knowledge about PrEP and lack of PrEP provision in settings where women seek sexual health care (9–18).

According to dissemination and implementation science, first steps to improve PrEP uptake among vulnerable populations include ensuring that those who can benefit from PrEP are aware of it, and ensuring PrEP is accessible in settings where they seek health care (19). The federally-funded Title X National Family Planning Program supports a nationwide network of ~4,000 family planning sites with over 3 million clients annually, 87% of whom are women (20). The program is designed to ensure contraception access, particularly for low-income youth and adults, but also funds preventive services including HIV testing and prevention. While most Title X clients are cis-gender women, clinics also serve men, transgender/gender nonconforming individuals, and youth, and therefore are poised to play an essential role in expanding PrEP access for multiple marginalized populations. For most clients, Title X clinics serve as their usual source of medical care, particularly in Southern states that have not expanded Medicaid (21, 22).

Despite being ideal sites for PrEP delivery, several studies have revealed that Title X clinics do not offer PrEP (9, 10, 23, 24), despite clinical guidelines having incorporated PrEP (25). Specific to clinics in the Southern U.S., Sales et al. surveyed nearly 600 providers/staff working in 286 Title X clinics across the South in 2018; only 22% of clinics provided any PrEP services, and the Southeastern region (including Alabama, Florida, Georgia, Kentucky, Mississippi, North Caroline, South Carolina, and Tennessee) had the fewest clinics offering PrEP (9, 26). Slow adoption of new evidence-based interventions, like PrEP, is a widespread concern in healthcare (27, 28). Organizations have difficulty implementing new interventions, often due to challenges coordinating change across a practice setting, rather than lack of recognizing the new intervention as relevant and desirable (27, 29). In line with the implementation science literature, Sales et al. also found that inner-setting factors from the Consolidated Framework for Implementation Research (CFIR) (30), such as having a climate supportive of HIV prevention interventions, supportive leadership, availability of resources, and individual attitudes about PrEP’s suitability for family planning were the salient factors associated with readiness to provide PrEP among clinics not doing so (31).

However, Title X clinics are part of a diverse network, with clinics operating within different social and policy environments (e.g., states with Medicaid expansion and/or PrEP Drug Assistance Programs); factors captured as part of the outer setting of CFIR. Although it is commonly acknowledged that outer setting factors such as state-level policies can impact clinic-level implementation, the outer setting is rarely considered in analyses of clinic-level implementation, and to our knowledge has not been explored explicitly in the context of PrEP implementation in the Southern U.S., a region with fewer PrEP-providing clinics relative to other regions nationally. The goal of this secondary analysis was to explore state-level clustering of organizational constructs relevant to PrEP implementation in Title X clinics in the Southern U.S.

## Methods

### Study design

From February–June 2018, we conducted a web-based, geographically-targeted quantitative survey of clinicians, staff, and administrators of publicly-funded family planning clinics not providing PrEP located in 18 U.S. states. Specifically, the survey was sent to Title X family planning clinics in DHHS regions III (Washington D.C., Delaware, Maryland, Pennsylvania, Virginia, and West Virginia), IV (Alabama, Florida, Georgia, Kentucky, Mississippi, North Carolina, South Carolina, Tennessee), and VI (Arkansas, Louisiana, New Mexico, Oklahoma, and Texas). The National Clinical Training Center for Family Planning (NCTCFP) supported our online recruitment of participants via listserv emails and advertisement on their website. Additional recruitment efforts included engagement with state Title X grantees and in-person recruitment at an NCTCFP national meeting.

Among 742 respondents from an eligible Title X DHHS region who agreed to participate in the survey, 519 (69.9%) completed the survey. Region IV (Southeast) had more respondents compared to III (Mid-Atlantic) and VI (Southwest) (329 (63.4%) vs. 126 (24.3%) and 64 (12.3%), respectively). Most respondents were clinic providers or support staff (436 (84.0%) vs. 83 (16.0%) administrators). Survey respondents represented 283 unique clinics across the three regions (30.7% in Region III, 54.4% in Region IV, and 14.8% in Region VI), with 76 (26.9%) of those clinics rurally-located. Only 59 (20.9%) clinics provided PrEP (33.3% of clinics from Region III provide PrEP, 14.3% of clinics from Region IV, and 19.0% of clinics from Region VI); only four PrEP providing clinics were rurally-located. Our secondary analyses presented here included 414 respondents from 224 clinics not providing PrEP. A comprehensive overview of the study’s protocol, data collection instruments, and primary statistical analysis methods has been published elsewhere (9, 26).

### Measures

The CFIR (30) informed construct selection, including Readiness to Implement PrEP and additional constructs previously associated with Readiness to Implement PrEP in the primary analysis of this survey (9, 26). These additional constructs included Inner Setting Constructs (Implementation Climate – General and HIV-related; Leadership Engagement – General and PrEP-specific, Available Resources – General and PrEP-specific) and Characteristics of Individuals (PrEP Knowledge; PrEP Attitudes – General, Positive, and Negative). All CFIR construct measures were scored as semi-continuous composite scores based on collections of related survey items. Each outcome, except for PrEP Knowledge, was derived as the mean of one or more Likert scale survey items that were identified as having high internal consistency based on Cronbach’s Alpha (8). PrEP Knowledge was derived as the number of correct responses identified by the respondent from a set of 5 questions. The survey and all items for each construct are fully available and reported elsewhere (9, 26).

### Analysis

Relevant constructs of interest were derived as composite scores. We analyzed each construct individually using linear mixed models (LMMs) with fixed effects for state, provider and clinic-level covariates, and a random effect for clinic to account for correlation among respondents from the same clinic. A principal component analysis (PCA) of the resulting construct-specific, state-level fixed effects was performed as a dimension reduction technique to address limitations based on the number of states for which we had sufficient data to estimate state-level effects (*N* = 12; excluded 6 states due to insufficient data, defined as less than 10 respondents). Principal components (PCs) were inputted into a K-means clustering analysis, with K specified to 3 clusters, to examine the extent of state-level clustering after adjusting for state, provider, and clinic-level covariates.

**Figure 1:**
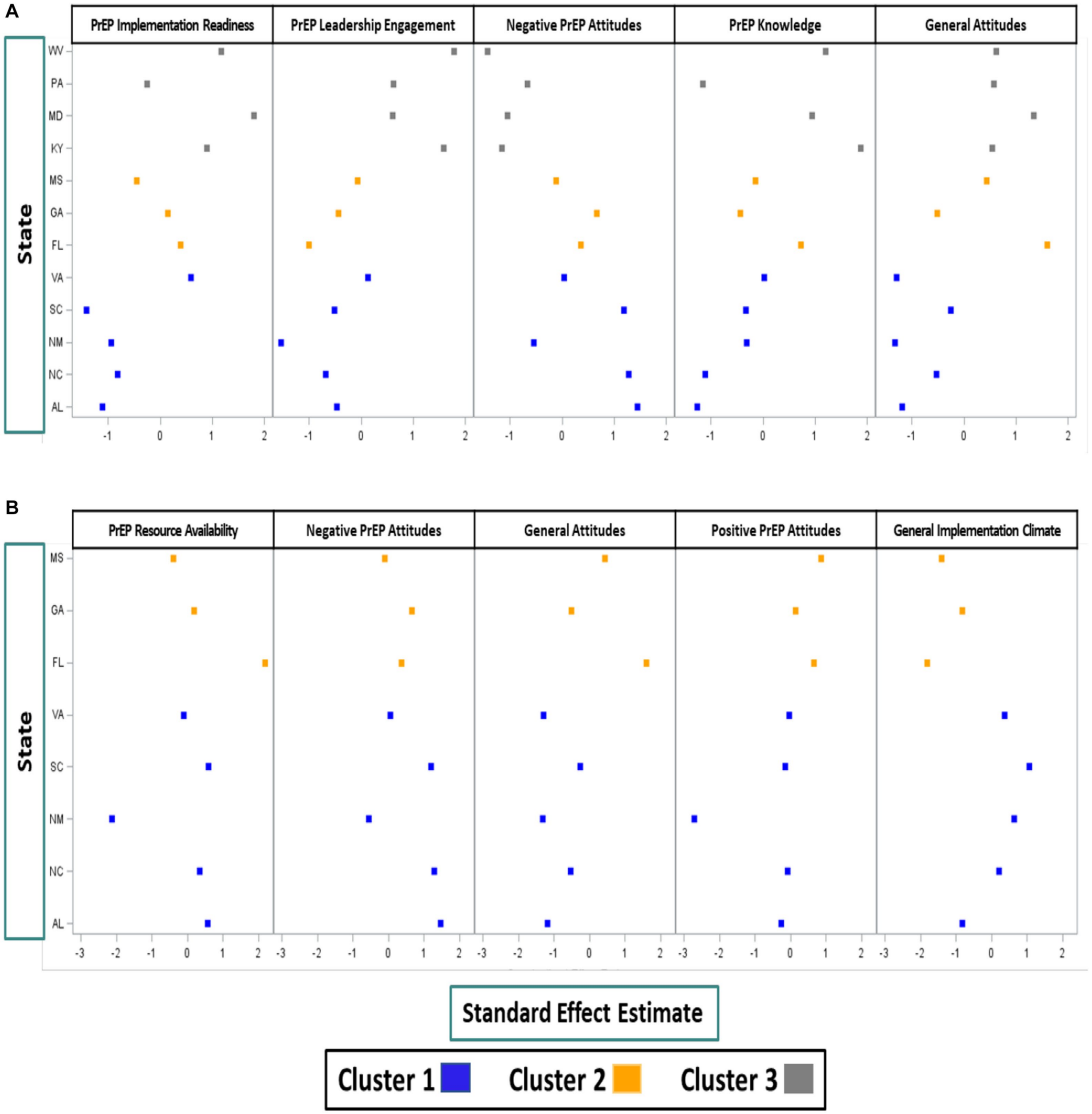
**(A)** Constructs most predictive of CV1 (distinguishing Cluster 3 from Cluster 1 and Cluster 2). **(B)** Constructs most predictive of CV2 (distinguishing Cluster 2 from Cluster 1).

To assess which constructs were important drivers of the clustering observed, we examined each construct’s contribution to a given canonical variable (CV) by summing the absolute value of the product of the construct weight in each of the six PCs and the weight of the corresponding PC in the cluster analysis. The constructs were then ranked separately for each of the two CVs from largest to least total weight. The five constructs contributing the largest total weight to each CV were then selected for further examination. Figures for each CV plot the standardized effect estimate of the mean state-level fixed effects for the five largest contributing constructs by state (Figures 1A,B). We grouped the resulting standardized effect estimates by cluster to assess directionality such that larger standardized effect estimates indicate larger values of the estimated state-level fixed effects for the construct of interest.

## Results

**Figure 2:**
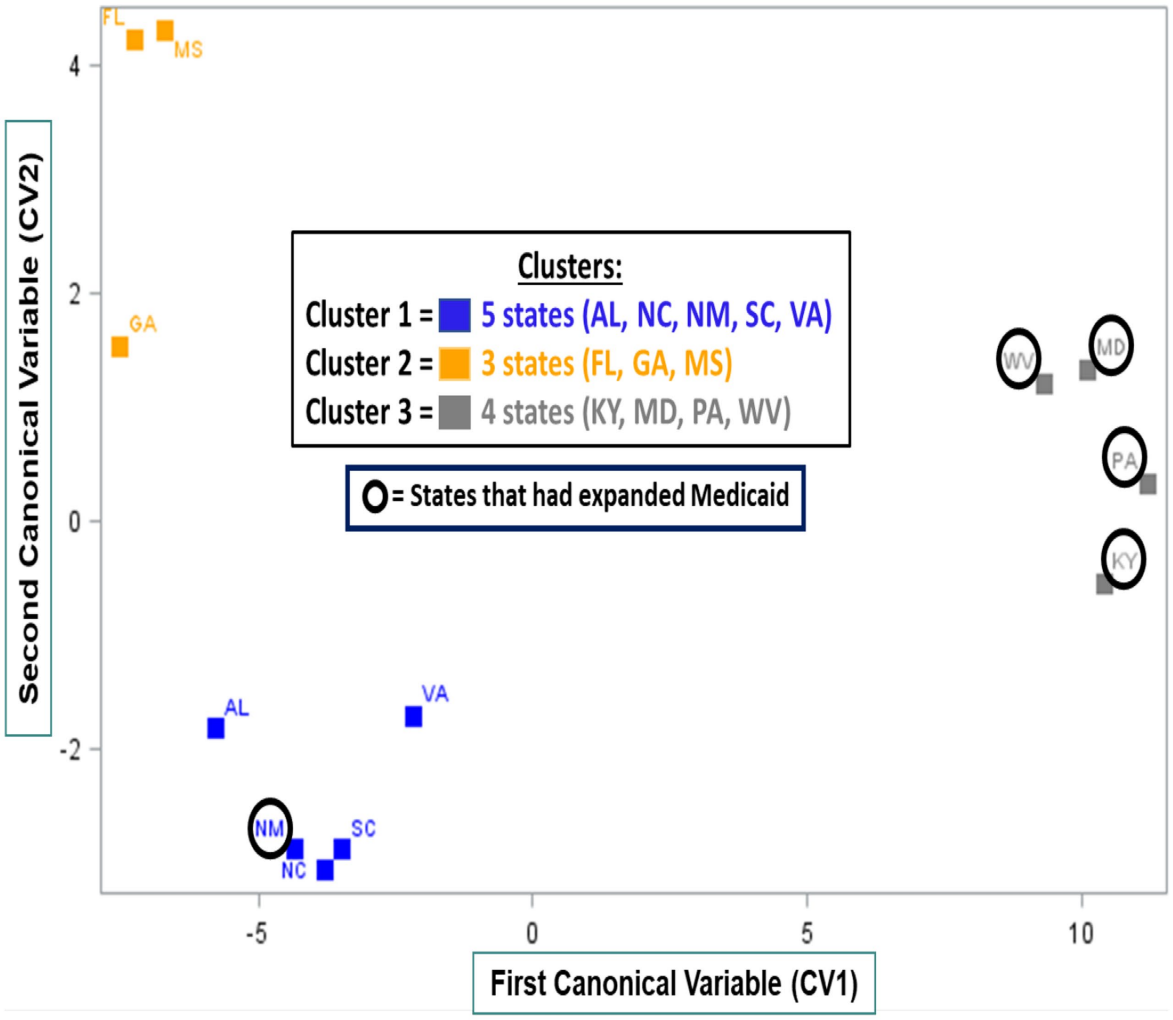
Clusters of 12 Southern US states stratified by first (CV1, x-axis) and second (CV2, y-axis) canonical variables derived from CFIR construct-specific, state-level fixed effect estimates.

Using the resulting estimates from the 12 LMM outcome models, the PCA identified six PCs that explained 96 percent of the variability in the estimated state-level construct effects. The K-means clustering analyses identified clusters of states with similar inner setting implementation constructs (Figure 2). The three clusters were characterized by five (Cluster 1: Alabama, New Mexico, North Carolina, South Carolina, and Virginia), three (Cluster 2: Florida, Georgia, and Mississippi), and four (Cluster 3: Kentucky, Maryland, Pennsylvania, and West Virginia) states, respectively. The first CV (CV1; x-axis) distinguished Cluster 3 from Cluster 1 and Cluster 2. Cluster 3 states were generally characterized by higher perceived PrEP implementation readiness, higher PrEP-specific leadership engagement, more favorable PrEP attitudes, and higher PrEP knowledge when compared to states from Cluster 1 and Cluster 2 (Figure 1A). All Cluster 3 states had expanded Medicaid by the time of the survey, compared to only one state in Cluster 1 and no states in Cluster 2.

The second CV (CV2; y-axis), distinguished Cluster 2 from Cluster 1. Cluster 2 states were characterized by higher perceived PrEP-specific resource availability, more favorable PrEP attitudes, and lower perceived general implementation climate when compared to Cluster 1 (Figure 1B).

## Discussion

Our study demonstrates that among Title X clinics in the Southern U.S. which were not providing PrEP, inner setting constructs identified as salient for PrEP implementation exhibited state-level clustering, thus suggesting outer setting factors’ potential impact on inner-setting PrEP implementation determinants. This secondary analysis spanned 12 states in DHHS regions III (Maryland, Pennsylvania, Virginia, West Virginia), IV (Alabama, Florida, Georgia, Kentucky, Mississippi, North Carolina, South Carolina), and VI (New Mexico). We observed noticeable clustering across the South, but state-level clustering of the 224 unique non-PrEP providing clinics in this sample was not observed within DHHS regions. Although states within DHHS regions may have similar geographical, social and policy environments, we observed that two of our clusters comprised states from different regions suggesting similar outer setting contextual factors among these states and that they may benefit from tailored strategies which could be used by clustered states to improve PrEP provision in Title X clinics. Improving PrEP access in places women seek sexual health care remains a critical priority in the South and will likely require attention to both outer and inner setting factors effecting PrEP implementation.

When considering salient outer setting features that differentiate our clusters from each other, Medicaid expansion was a common feature in Cluster 3 states, which were characterized by higher perceived readiness for PrEP implementation and other factors associated with PrEP implementation readiness/implementation (24). Successful adoption of new evidence-based interventions into healthcare settings has been characterized by several organizational factors, including provider/staff and administrators’ readiness to provide the new intervention (to what degree is it possible), their attitudes about the new intervention individually (is it desirable) as well as collectively (climate supportive of new intervention), leadership support (making the change a priority), and adequacy of resources (training, staffing, and financial) (32). When these factors are present before the adoption of a new intervention, they may indicate an organization’s readiness to adopt/implement the new intervention, and when collectively present, these factors have predicted successful implementation (32–34). Thus, Title X clinics in Cluster 3, with higher levels of readiness for PrEP implementation, higher PrEP supportive attitudes and greater PrEP knowledge compared to Clusters 1 and 2, may require relatively few implementations strategies to move them to PrEP implementation. However, Title X clinics in Clusters 1 and 2 with lower PrEP readiness may require more robust, time-intensive interventions to address challenges with organizational climate, leadership engagement, and more substantial resource constraints (e.g., staffing) identified as important for PrEP implementation in other studies (9, 24).

Commonly cited barriers to PrEP implementation included cost and lack of resources including training, staffing and time for providing PrEP (24, 35–40). Title X funding is allocated at the state-level (16). The state-level Title X grantee(s) distributes Title X funds to clinical service sites to support provision of family planning and preventive services, as well as provide training and technical assistance to clinical sites as they provide covered services. Several federal agencies have been working in a coordinated manner as part of the EHE initiative which has prioritized certain counties or states for receipt of additional funding to address HIV based on epidemiologically determined need in these communities (4), In states like those in Cluster 2, none of which have expanded Medicaid and all have been geographically prioritized by EHE, the provision of additional funds dedicated for PrEP delivery to the Title X programs of these states could improve PrEP delivery and ultimately PrEP reach to the low income, mostly minority women served by the Title X clinical sites in these states. Given that PrEP attitudes among providers and staff in these clinical sites were high, state grantee(se) could use these earmarked funds to incentivize clinical sites to provide PrEP and help them overcome any education and cost/resource barriers likely driving low climate for adopting new interventions.

The parent study was not designed to systematically assess outer setting factors pertaining to the geographic, social or policy environments of each state. However, the state-level clustering we observed for PrEP implementation in safety-net family planning clinics across a region of the country where some of the starkest racial HIV disparities exist for both men and women warrants further examination of the role of Medicaid expansion and other state-level policies (e.g., HIV criminalization, abortion bans, anti-LGBTQ laws) on PrEP provision.

## Limitations

Our study has several limitations. Ours was a convenience sample of clinic providers and administrators, thus may be subject to selection bias. Clinic characteristics were provided by self-report rather than direct observations. Finally, the study was conducted among staff of Title X funded-family planning clinics, and therefore findings may not be generalizable across other women’s health settings. Nonetheless, a key strength of this study was the large sample size, along with the diversity of geographic location and clinic characteristics among the clinics represented by study participants.

## Conclusion

The Title X family planning program is a vital safety-net clinical network providing sexual health care for millions of low-income individuals across the U.S., including many minoritized and marginalized populations (i.e., Black women and LGBTQ individuals). For many women, especially in states that did not expand Medicaid, Title X clinics serve as their sole source of health care. Despite this, there has been limited discussion of the role of this vital safety net in achieving the ambitious targets set forth by the EHE initiative. Given persistent health disparities in the U.S., the EHE initiative should leverage and expand on the important role that this network of family planning clinics continues to play in providing HIV testing and preventive services to the 3 million people they annually serve. Our study indicates that clinic-level barriers and facilitators to providing PrEP cluster across states, suggesting that salient social and policy-related outer setting factors may be associated with clinic-level inner setting determinants to providing PrEP in these otherwise ideal PrEP-delivery sites for women. Greater attention is needed to focus implementation strategies at multiple levels of the social ecology, including policy drivers of heath inequities, to improve PrEP access and ultimately PrEP reach among Black women in the Southern U.S.

## Data availability statement

The raw data supporting the conclusions of this article will be made available by the authors, without undue reservation.

## Ethics statement

The studies involving human participants were reviewed and approved by Emory University Institutional Review Board. The patients/participants provided their written informed consent to participate in this study.

## Author contributions

JS and AS conceptualized this study, drafted and edited the manuscript. MM, KE, and MP conducted statistical analysis and contributed to the drafting of the manuscript. AR contributed to the drafting and editing of the manuscript. All authors contributed to the article and approved the submitted version.

## Funding

This study was funded by an NIH-funded grant to JS and AS as part of the Adolescent Medicine Trials Network for HIV/AIDS Interventions (ATN), U24HD089880, and the Emory Center for AIDS Research (P30AI050409).

## Conflict of interest

The authors declare that the research was conducted in the absence of any commercial or financial relationships that could be construed as a potential conflict of interest.

## Publisher’s note

All claims expressed in this article are solely those of the authors and do not necessarily represent those of their affiliated organizations, or those of the publisher, the editors and the reviewers. Any product that may be evaluated in this article, or claim that may be made by its manufacturer, is not guaranteed or endorsed by the publisher.
